# Comparison of quality of life between Billroth-І and Roux-en-Y anastomosis after distal gastrectomy for gastric cancer: A randomized controlled trial

**DOI:** 10.1038/s41598-017-09676-2

**Published:** 2017-09-12

**Authors:** Kun Yang, Wei-Han Zhang, Kai Liu, Xin-Zu Chen, Zong-Guang Zhou, Jian-Kun Hu

**Affiliations:** 1 Department of Gastrointestinal Surgery, West China Hospital, Sichuan University, Chengdu, China; 2 Laboratory of Gastric Cancer, State Key Laboratory of Biotherapy/Collaborative Innovation Center of Biotherapy and Cancer Center, West China Hospital, Sichuan University, Chengdu, China

## Abstract

Studies comparing Billroth-I (B-I) with Roux-en-Y (R-Y) anastomosis are still lacking and inconsistent. The aim of this trial was to compare the quality of life (QoL) of B-I with R-Y reconstruction after curative distal gastrectomy for gastric cancer. A total of 140 patients were randomly assigned to the B-I group (N = 70) and R-Y group (N = 70) with the comparable baseline characteristics. The overall postoperative morbidity rates were 18.6% and 25.7% in the B-I group and R-Y group without significant difference. More estimated blood loss and longer surgical duration were found in the R-Y group. At the postoperative 1 year time point, the B-I group had a higher score in pain, but lower score in global health. However, the R-Y anastomosis was associated with lower incidence of reflux symptoms at postoperative 6 months (P = 0.002) and postoperative 9 months (P = 0.007). The multivariable analyses of variance did not show any interactions between the time trend and grouping. For the results of endoscopic examination, the degree and extent of remnant gastritis were milder significantly in the R-Y group. The stronger anti-reflux capability of R-Y anastomosis contributes to the higher QoL by reducing the reflux related gastritis and pain symptoms, and promotes a better global health.

## Introduction

Gastric cancer remains a disease with high incidence and is responsible for about 10% of all cancer-related deaths in the world^1^. Although the incidence of gastric cancer at the upper third of stomach has gradually increased over the years, distal gastric cancers as well as distal gastrectomy are still the mainstream^2^. In recent years, the overall survival of patients with gastric cancer has improved as the increase of proportion of early gastric cancer detection, the implementation of standard D2 lymphadenectomy, the development of chemotherapy and new targeted drugs^3–5^. Accompanied with the improved survival, the quality of life (QoL) has attracted more attentions than before. One of the most associated factors with QoL of patients after gastrectomy is the type of digestive tract reconstruction.

Billroth-I (B-I) and Roux-en-Y (R-Y) reconstruction after distal gastrectomy have been widely applied. Both B-I and R-Y have their own advantages and disadvantages. B-I has merits of technical simplicity and preservation of physiological food passage. However, patients undergoing B-I reconstruction frequently suffer from the reflux symptoms, which could cause remnant gastritis and esophagitis, even increase the possibility of remnant gastric cancer or esophageal cancer^6, 7^. In the contrast, R-Y reconstruction has strong capacity to prevent bile reflux theoretically^8^. Nevertheless, patients with R-Y reconstruction often complain so-called stasis syndrome, which has the significant postoperative symptoms including abdominal pain, nausea, and vomiting^9, 10^. Furthermore, the difficulty of postoperative duodenal endoscopic examination makes surgeons reluctant to perform R-Y reconstruction.

Currently, although a few studies have compared the morbidity, mortality and QoL of B-I with R-Y anastomosis, their findings are still inconsistent^11–16^. Moreover, most of the studies which compared B-I with R-Y anastomosis were carried out in Japan and Korea where the proportions of early gastric cancer are more than 50%^17^. Therefore, the generalizability of such evidence to other countries where most of the patients has advanced gastric cancer still needs addressing. In addition, there was no study to dynamically compare the QoL of B-I with R-Y reconstruction by standard questionnaires at multiple time points.

Therefore, we performed a prospective randomized controlled trial (RCT) to compare B-I with R-Y reconstruction after curative distal gastrectomy for gastric cancer in China. Here, we reported the results of 1-year interim analysis.

## Materials and Methods

### Patients

The inclusion criteria were the following: patients aged 18 to 75 with tolerance of the operation; the score of World Health Organization performance status being less than 2; preoperative diagnosis of gastric adenocarcinoma was confirmed by gastric endoscopy and biopsy; the tumors located at lower third of stomach for which distal gastrectomy was feasible; the preoperative staging of potential curative resectable tumors was less than T4aN2M0 according to the 3^rd^ English edition of Japanese Classification of Gastric Carcinoma^18^; either open gastrectomy or laparoscopic assisted gastrectomy was included. The exclusion criteria consisted of: patients with history of previous laparotomy (except appendectomy and laparoscopic cholecystectomy); patients needed receiving total gastrectomy or combined organ resection (except cholecystectomy) for curative purpose; patients were diagnosed with other gastric malignances, such as lymphoma and gastrointestinal stromal tumor *etc*, any previous malignancies or synchronous malignancies; emergency cases because of perforation or bleeding of tumor; patients had neo-adjuvant chemotherapy or perioperative radiotherapy.

This study was registered on Chinese Clinical Trial Register (ChiCTR), WHO (SN. ChiCTR-TRC-10001434, date of registration: November 24^th^, 2010), and approved by the West China Hospital research ethics committee, and the study was done in accordance with the Declaration of Helsinki. Written informed consent were obtained from all patients.

### Study design

After being generated by random number table, the random allocation sequence was concealed and sealed in sequential numbered and opaque envelopes, which were uncovered following the initial laparotomy to assess the eligibility of patients. Then, included patients were randomly assigned to the B-I or R-Y group in a 1:1 ratio intraoperatively. In this study, patients, surgeons, staffs who collected data and analyzed outcomes were not blinded. During the study period, the study protocol was not amended.

### Surgical technique

The nasogastric tube was placed routinely before the operation. All the patients underwent distal gastrectomy with D2 lymphadenectomy according to the Japanese gastric cancer treatment guideline^19^. All the anastomoses were completed by using the mechanical staplers and reinforced by interrupted full-thickness sutures. Briefly, the end-to-side gastroduodenostomy was made by 25 mm circular stapler between the duodenal stump and posterior wall of the remnant stomach in the B-I group. And the gastric stump was closed by linear stapler. In the R-Y group, the duodenum was divided and closed by a linear stapler 3 cm distal to the pylorus. Then, the duodenal stump was reinforced by interrupted full-thickness sutures and interrupted seromuscular sutures. For reconstruction, the proximal jejunum was identified and divided 15–20 cm distant from the Treitz ligament firstly. Next, the gastrojejunostomy was performed between posterior wall of the remnant stomach and antimesenteric border of the distal jejunums with a 25 mm circular stapler in a side-to side fashion. Then, a side-to-side jejunojejunostomy was created between the antimesenteric borders of the proximal and distal jejunums 45 cm below the gastrojejunostomy. The gastric stump and two jejunal stumps were closed by linear staplers. The mesenteric defect was closed with interrupted sutures.

In the patients who received laparoscopic surgery, we performed laparoscopic assisted distal gastrectomy. After the lymph nodes dissection by using laparoscopy, a mini-incision at length of 5–8 cm was made above the umbilicus. Then specimen was removed and extracorporeal digestive tract reconstruction was performed through the incision. The procedures and staplers used for specimen removal and anastomosis were as same as the open cases.

The drain was also placed routinely through the Winslow foramen. All the surgeons adhered with the protocol. The adherence and the quality of operation were evaluated by the study group through scanning the intraoperative photos.

### Postoperative care protocol

After the operation, a standardized postoperative care protocol was applied. Intravenous antibiotic prophylaxis and parenteral nutritional support were given to all patients. The nasogastric tube was removed after the first gas-passing, and sips of water started thereafter. Liquid diet and soft diet were given over the next 2 days gradually. The drain was usually removed on postoperative day 5 if there were no complications. If there was no complications after 1 day of soft diet, discharge of patients was encouraged. If there were any compilations, treatments were given individually.

### Outcome measurements

The primary end point of this RCT was QoL. The data of QoL was collected by the validated European Organization for Research and Treatment of Cancer (EORTC) core questionnaire (EORTC QLQ-C30, version 3.0), and stomach module questionnaire (EORTC QLQ-STO22)^20^. The EORTC QLQ-C30 questionnaire contains 30 items, which could be used to assess the QoL of all kinds of cancer patients. The 30 items could be incorporated into five functional scales (physical, role, cognitive, emotional, and social), three symptom scales (pain, fatigue, nausea and vomiting), six single items (dyspnea, insomnia, appetite loss, constipation, diarrhea and financial difficult) and one global health status scale. Except for the global health scale in which the item values range from 1 to 7, other items are scored 1 to 4, corresponding to the four response categories, namely “Not at all”, “A little”, “Quite a bit” and “Very much”^21^. The EORTC QLQ-STO22 questionnaire comprises 22 items, which could be divided into five symptom scales (dysphagia, pain, eating restrictions, reflux symptoms, and anxiety) and four single items (having a dry mouth, taste, body image, and hair loss)^22^. A high score represents a high/healthy level of functioning or QoL for a functional scale or the global health status; whereas a high level of symptomatology/problems for symptom scales/items^21^. The preoperative baseline of QoL was obtained 3 days before the operation. Postoperative QoL, recurrence and survival status were collected every 3 months after the operation by telephone calls, letters or outpatient visits. No patient was lost to follow-up.

The secondary end points were operative safety, postoperative recovery and severity of postoperative gastritis. Clinicopathologic terminology was based on the 3^rd^ English edition of Japanese Classification of Gastric Carcinoma^18^. Morbidity, spectrum of complications and mortality were compared. The complications were classified according to the Clavien-Dindo Classification^23^. The estimated blood loss, operative duration, postoperative hospital stay, mean time to the first flatus and mean time to the first food intake were also compared. The severity of postoperative gastritis was evaluated by endoscopic examination at postoperative 12 months and graded according to the “residue, gastritis, bile” classification^24^.

### Statistical analyses

The sample size was calculated by using a two-sided alpha error of 5% under the normal distribution with a standard deviation of 0.1. And the effect size was determined according to Kojima’s study which showed that the heartburn rates of the B-I group and R-Y group were 37% and 8% at the postoperative 1 year respectively^15^. The planned sample size was calculated as 56 in each arm. Finally, we enrolled 70 patients in each arm, allowing for a 15–20% dropout rate.

SPSS 19.0 software (SPSS, Chicago, IL, USA) was used for statistical analysis. All QLQ-C30 and QLQ-STO22 responses were linearly transformed to scores from 0 to 100 according to EORTC scoring manuals^21^. Quantitative data was expressed as means ± standard deviation (SD) and tested by One-way ANOVA test. For categorical data, the Chi-square was used to compare frequencies. Non-parametric tests were performed for non-normal distribution data. Multivariable analysis of variance was applied to investigate the interaction of time and grouping. A P value of less than 0.05 (two-sided) was considered statistically significant. An intention-to-treat analysis was applied as the main statistical method to avoid potential biases.

## Results

### Characteristics of patients

**Figure 1:**
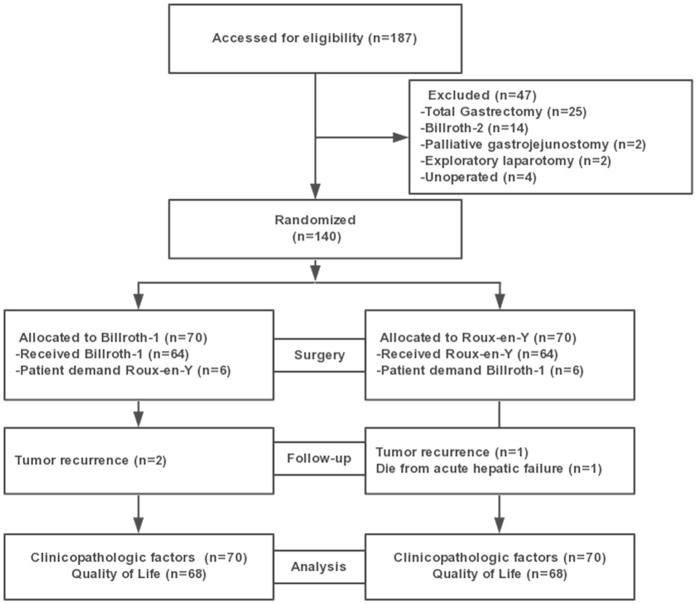
Trial profile.

From May, 2011 to May, 2014, a total of 140 gastric cancer patients who underwent distal gastrectomy were randomly assigned to the B-I group (N = 70) and R-Y group (N = 70). In B-I group, there were 6 patients converting to the R-Y anastomosis because of the presence of tension between the duodenum and remnant stomach. In R-Y group, 6 patients underwent the B-I anastomosis due to the willingness or economical consideration of patients’ families. As of postoperative 1 year, 2 patients in the B-I group and 1 patient in the R-Y group recurred, 1 patient in the R-Y group died due to chemotherapy associated hepatic failure. Figure 1 shows the trial profile. The baseline characteristics of patients were comparable between the two groups (Table 1).

**Table 1:** Clinicopathological characteristics of patients in the two groups

Variables	B-I group N = 70 (%)	R-Y group N = 70 (%)	P Value
**Gender**			0.223
Male	40 (57.1)	47 (67.1)	
Female	30 (42.9)	23 (32.9)	
**Age**	56.3 ± 10.7	54.9 ± 11.5	0.491
**Educational Level**			0.111
<7 years	26 (37.1)	17 (24.3)	
7–12 years	29 (41.4)	28 (40.0)	
>12 years	15 (21.4)	25 (35.7)	
**BMI (kg/m2)**	22.4 ± 3.1	22.7 ± 3.5	0.616
**Tumor size (cm)**	3.6 ± 1.9	4.4 ± 2.6	0.156
**Macroscopic type**			0.338
Type 0-2	54 (77.1)	49 (70.0)	
Type 3-4	16 (22.9)	21 (30.0)	
**Histologic Type**			0.494
Well and Moderate differentiated	13 (18.6)	10 (14.3)	
Poor and undifferentiated	57 (81.4)	60 (85.7)	
**Lauren classification**			0.178
Intestinal type	27 (38.6)	22 (31.4)	
Diffuse type	38 (54.3)	36 (51.4)	
Mixed type	5 (7.1)	12 (17.1)	
**Surgical Type**			0.699
Open Surgery	53 (75.7)	51 (72.9)	
Laparoscopic Surgery	17 (24.3)	19 (27.1)	
**No. of positive LNs**	4.6 ± 7.6	4.6 ± 7.0	0.917
**No. of examined LNs**	32.9 ± 12.5	36.6 ± 18.2	0.173
**Depth of invasion**			0.303
T1	27 (38.6)	22 (31.4)	
T2	12 (17.1)	11 (15.7)	
T3/ T4	31 (44.3)	37 (52.9)	
**Lymph nodes metastasis**			0.764
N0	27 (38.6)	30 (42.9)	
N1	13 (18.6)	10 (14.3)	
N2	15 (21.4)	12 (17.1)	
N3	15 (21.4)	18 (25.7)	
**TNM stage**			0.237
IA	18 (25.7)	13 (18.6)	
IB	12 (17.1)	9 (12.9)	
IIA	8 (11.4)	8 (11.4)	
IIB	6 (8.6)	11 (15.7)	
IIIA	10 (14.3)	6 (8.6)	
IIIB	3 (4.3)	10 (14.3)	
IIIC	10 (14.3)	9 (12.9)	
IV	3 (4.3)	4 (5.7)	
**Adjuvant Chemotherapy**			0.062
No	37 (52.9)	26 (37.1)	
Yes	33 (47.1)	44 (62.9)	

### Operative variable and Postoperative recovery

There was no perioperative mortality. The overall postoperative morbidity rates were 18.6% (13/70) and 25.7% (18/70) in the B-I group and R-Y group without significant difference (P = 0.309). Postoperative pulmonary infection was the most frequent complication in both groups. There was no anastomosis-related complications. The constitution of complications between the two groups had no significant difference. The estimated blood loss and surgical duration were significantly less in the B-I group. There were no significant differences in terms of postoperative hospital stay, time to first gas-passing, time to first oral intake, time to nasogastric decompression removal and time to drain removal between the two groups. The details can be seen in Table 2.

**Table 2:** Operative safety and postoperative recovery between the two groups.

	B-I group N = 70 (%)	R-Y group N = 70 (%)	P Value
Mortality	0	0	1.000
Morbidity	13 (18.6)	18 (25.7)	0.309
Clavien-Dindo classification			0.921
I	2 (15.4)	4 (22.2)	
II	11 (84.6)	13 (72.2)	
IIIa	0	0	
IIIb	0	1 (5.6)	
IVa	0	0	
IVb	0	0	
V	0	0	
Postoperative Complications			
Pulmonary complications	10	10	1.000
Acute cholecystitis	1	1	1.000
Superficial surgical site infection	0	2	0.496
Intra-abdominal infection	0	2	0.496
Adhesive ileus	0	1	1.000
Acute urinary retention	1	1	1.000
Gastroplegia	1	1	1.000
Surgical duration (min)	239.4 ± 40.8	271.2 ± 39.2	<0.001
Estimated blood loss (ml)	84.1 ± 32.0	104.2 ± 35.3	<0.001
Postoperative hospital stay (days)	9.6 ± 1.2	10.3 ± 3.7	0.674
Time to nasogastric tube removal (days)	4.3 ± 1.4	4.3 ± 1.3	0.936
Time to drain removal (days)	6.9 ± 1.2	7.6 ± 3.8	0.227
Time to first gas-passing (days)	4.7 ± 1.1	4.6 ± 0.9	0.724
Time to first oral intake (days)	5.8 ± 1.1	5.7 ± 1.3	0.664

### Assessment of QoL

**Figure 2:**
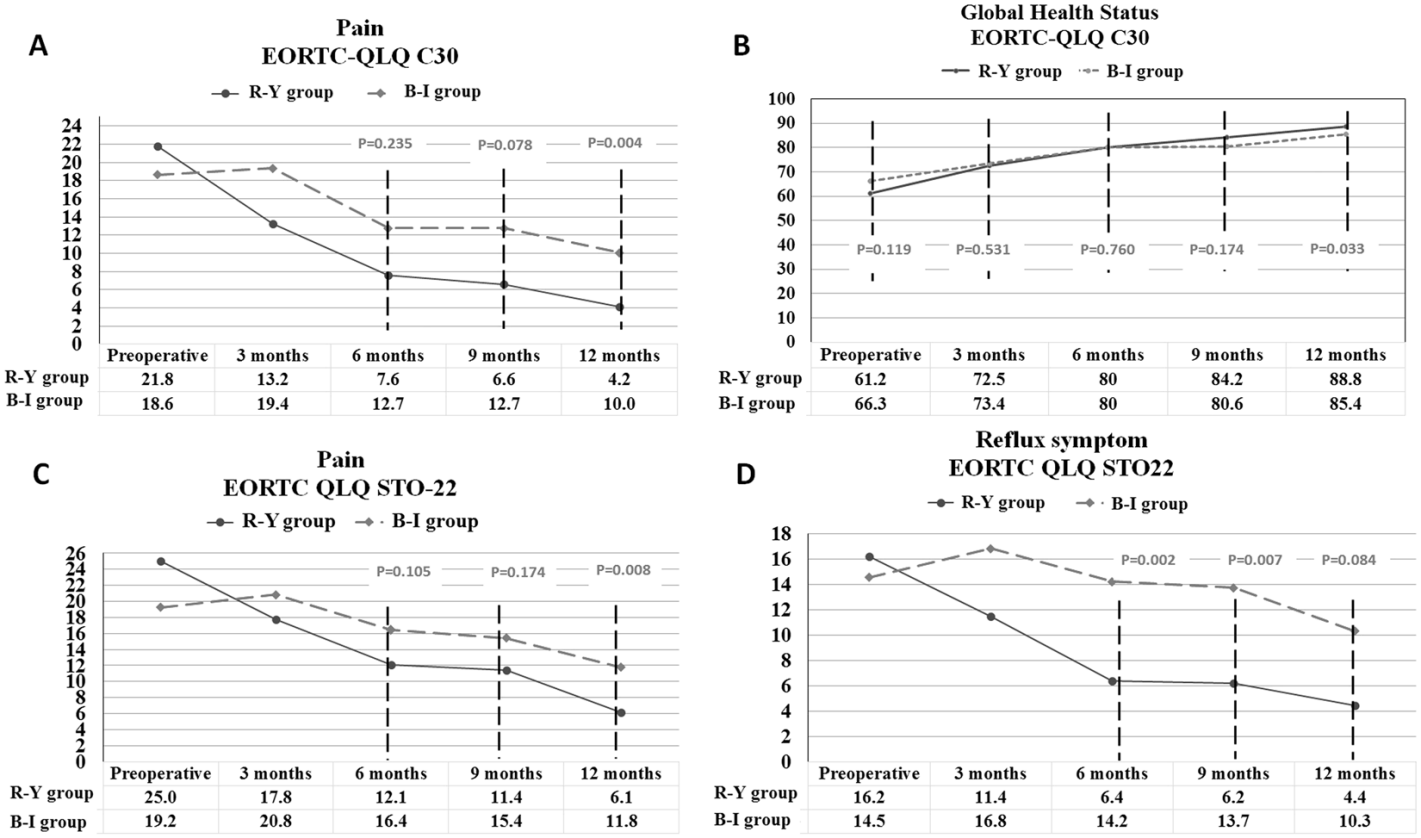
Comparison of QoL scores from preoperative baseline to postoperative 1 year between the two groups. (**A**) Pain item in QLQ-C30 questionnaire; (**B**) Global Health Status in QLQ-C30 questionnaire; (**C**) Pain item in QLQ-STO22 questionnaire; (**D**) Reflux symptom item in QLQ-STO22 questionnaire.

Except for the insomnia item, there was no significant difference of baseline QoL between the groups (Table 3). With respect to the EORTC QLQ-C30 items, no significant differences were identified between the two groups at the postoperative 3 months and 6 months. For insomnia, a better outcome was observed in the B-I group with difference about 7 points at postoperative 9 months; however, this difference turned to not significant at postoperative 1 year. At the postoperative 1 year time point, a higher score of global health status and a lower score of pain were detected in the R-Y group compared with the B-I group, which showed an overall average difference of 3 points and 6 points respectively. And R-Y group had showed a better trend for the dyspnea item without significant difference. The other scales, such as physical functioning, fatigue, diarrhea, and financial difficulties, were not significantly different between the two groups (Table 3, Fig. 2).

**Table 3:** The preoperative and postoperative QoL of patients between the two groups according to the EORTC QLQ-C30 items.

**Items**	**Preoperative**			**Postoperative 3 months**			**Postoperative 6 months**			**Postoperative 9 months**			**Postoperative 1 year**		
	**B-I group**	**R-Y group**	**P**	**B-I group**	**R-Y group**	**P**	**B-I group**	**R-Y group**	**P**	**B-I group**	**R-Y group**	**P**	**B-I group**	**R-Y group**	**P**
Physical functioning	93.1 ± 7.9	93.4 ± 8.3	0.710	92.4 ± 11.8	91.3 ± 10.8	0.430	95.2 ± 8.8	96.6 ± 6.9	0.356	93.9 ± 9.5	96.3 ± 8.8	0.073	96.7 ± 7.3	97.5 ± 7.5	0.339
Role functioning	93.4 ± 12.9	91.4 ± 16.7	0.966	90.4 ± 15.9	90.4 ± 15.3	0.916	94.4 ± 13.1	94.9 ± 12.6	0.929	92.4 ± 13.3	94.4 ± 11.4	0.459	96.3 ± 10.3	99.0 ± 4.0	0.124
Cognitive functioning	89.2 ± 17.5	90.9 ± 13.0	0.795	96.8 ± 8.8	97.3 ± 7.9	0.932	96.1 ± 10.0	98.0 ± 7.4	0.121	95.3 ± 12.2	96.8 ± 10.1	0.448	97.1 ± 10.0	98.3 ± 7.1	0.379
Emotional functioning	83.9 ± 20.0	83.1 ± 15.4	0.319	96.2 ± 8.7	95.3 ± 10.2	0.773	96.2 ± 8.7	97.1 ± 7.6	0.613	94.7 ± 11.6	96.1 ± 9.7	0.502	95.2 ± 11.2	96.4 ± 8.6	0.857
Social functioning	86.3 ± 20.3	85.3 ± 19.9	0.799	95.3 ± 10.3	95.1 ± 12.6	0.621	96.3 ± 10.7	97.1 ± 10.8	0.572	96.8 ± 9.7	95.6 ± 14.0	0.767	98.0 ± 6.8	97.3 ± 12.4	0.557
Global Health status	66.3 ± 27.3	61.2 ± 23.8	0.119	73.4 ± 21.5	72.5 ± 17.9	0.531	80.0 ± 20.3	80.0 ± 17.8	0.760	80.6 ± 18.3	84.2 ± 18.3	0.174	85.4 ± 13.1	88.8 ± 16.1	0.033
Pain	18.6 ± 20.5	21.8 ± 17.1	0.143	19.4 ± 25.0	13.2 ± 15.9	0.382	12.7 ± 18.5	7.6 ± 11.7	0.235	12.7 ± 19.1	6.6 ± 11.9	0.078	10.0 ± 13.9	4.2 ± 9.7	0.004
Fatigue	17.0 ± 17.6	16.0 ± 16.6	0.814	13.2 ± 14.1	11.3 ± 13.4	0.401	10.5 ± 14.5	10.0 ± 14.1	0.757	11.3 ± 16.0	8.8 ± 13.5	0.340	9.3 ± 14.9	7.0 ± 13.3	0.319
Nausea and vomiting	9.6 ± 19.8	10.3 ± 15.0	0.395	6.4 ± 12.2	6.1 ± 11.1	0.839	2.0 ± 6.1	3.7 ± 8.6	0.207	5.4 ± 15.1	4.4 ± 10.6	0.852	2.7 ± 8.9	2.0 ± 6.8	0.752
Insomnia	19.6 ± 23.9	8.8 ± 15.9	0.004	8.8 ± 16.9	5.9 ± 14.0	0.277	9.8 ± 19.2	6.4 ± 16.5	0.253	12.3 ± 6.4	19.9 ± 16.5	0.031	8.3 ± 16.7	3.9 ± 12.3	0.064
Appetite loss	11.3 ± 22.0	12.3 ± 19.0	0.487	6.9 ± 13.6	9.3 ± 17.1	0.488	8.3 ± 16.7	4.9 ± 13.2	0.176	5.9 ± 14.0	4.4 ± 14.0	0.341	4.9 ± 13.2	2.5 ± 10.5	0.153
Constipation	7.8 ± 14.2	10.8 ± 18.6	0.470	6.9 ± 15.8	3.4 ± 11.7	0.131	3.4 ± 11.7	2.5 ± 8.8	0.737	4.4 ± 15.1	3.4 ± 10.2	0.841	2.9 ± 11.1	1.5 ± 6.9	0.458
Diarrhea	8.3 ± 15.6	11.8 ± 19.8	0.322	7.8 ± 17.4	11.8 ± 19.8	0.173	10.3 ± 21.7	9.8 ± 20.8	0.966	6.9 ± 14.7	6.4 ± 14.4	0.828	6.9 ± 14.7	7.8 ± 19.2	0.897
Dyspnea	3.4 ± 10.2	4.4 ± 11.4	0.596	6.4 ± 16.5	2.9 ± 9.5	0.259	4.9 ± 15.5	2.5 ± 10.5	0.336	6.4 ± 14.4	3.9 ± 13.5	0.149	3.4 ± 11.7	0.5 ± 4.0	0.053
Financial difficulties	22.1 ± 31.9	21.6 ± 28.1	0.822	5.9 ± 15.2	6.9 ± 16.8	0.673	4.9 ± 11.9	4.9 ± 15.5	0.641	5.4 ± 12.4	5.4 ± 16.9	0.510	8.8 ± 20.5	4.9 ± 16.6	0.149

The scores of physical functioning, role functioning and global health status increased; while the scores of pain, nausea and vomiting showed decreased trends from the postoperative 3 months to the postoperative 1 year. However, there were no significant variations of other scales accompanied with the time trend. The multivariable analyses of variance did not show any interactions between the time trend and grouping, which meant that the variation of each scale accompanied with the time trend did not depend on the grouping (Fig. S1, supporting information).

Regarding to the QLQ-STO22 items, R-Y anastomosis was associated with lower incidence of reflux symptoms at postoperative 6 months (P = 0.002) and postoperative 9 months (P = 0.007). However, these differences discontinued to postoperative 1 year. A significant lower score of pain was noted in the R-Y group at postoperative 1 year (P = 0.008). However, there were no significant differences on symptoms such as dysphagia, eating restrictions, having a dry mouth, change of taste, anxiety, body image and hair loss (Table 4, Fig. 2).

**Table 4:** The preoperative and postoperative QoL of patients between the two groups according to the EORTC QLQ-STO22 items.

**Items**	**Preoperative**			**Postoperative 3 months**			**Postoperative 6 months**			**Postoperative 9 months**			**Postoperative 1 year**		
	**B-I group**	**R-Y group**	**P**	**B-I group**	**R-Y group**	**P**	**B-I group**	**R-Y group**	**P**	**B-I group**	**R-Y group**	**P**	**B-I group**	**R-Y group**	**P**
Dysphagia	4.7 ± 9.5	5.6 ± 10.2	0.736	6.4 ± 12.6	7.0 ± 11.2	0.529	4.2 ± 11.4	3.4 ± 7.5	0.993	6.4 ± 11.6	4.4 ± 9.6	0.481	3.1 ± 8.3	3.4 ± 9.6	0.861
Pain	21.4 ± 15.3	25.0 ± 14.0	0.160	20.8 ± 19.5	17.8 ± 16.5	0.348	16.4 ± 15.8	12.1 ± 12.9	0.105	15.4 ± 17.8	11.4 ± 14.5	0.174	11.8 ± 12.7	6.1 ± 8.8	0.008
Reflux symptom	14.5 ± 19.1	16.2 ± 15.4	0.218	16.8 ± 20.6	11.4 ± 15.0	0.262	14.2 ± 16.9	6.4 ± 11.2	0.002	13.7 ± 16.2	6.2 ± 10.8	0.007	10.3 ± 15.0	4.4 ± 6.7	0.084
Eating restrictions	8.8 ± 13.4	8.9 ± 11.5	0.651	5.9 ± 10.1	6.3 ± 10.9	0.878	6.3 ± 11.2	3.2 ± 6.9	0.094	5.8 ± 11.1	4.2 ± 9.2	0.487	2.9 ± 7.0	2.3 ± 5.7	0.772
Having a dry mouth	16.2 ± 23.4	19.6 ± 21.0	0.192	4.4 ± 11.4	7.8 ± 16.4	0.231	7.4 ± 15.1	5.4 ± 12.4	0.486	6.9 ± 13.6	8.3 ± 17.6	0.802	6.9 ± 15.8	6.9 ± 18.7	0.688
Taste	8.8 ± 17.9	5.9 ± 14.0	0.291	6.4 ± 14.4	7.8 ± 15.4	0.527	6.9 ± 17.8	5.4 ± 12.4	0.940	5.9 ± 15.2	8.3 ± 16.7	0.286	6.4 ± 13.2	5.9 ± 17.2	0.402
Anxiety	17.8 ± 20.3	18.6 ± 19.7	0.650	6.7 ± 12.3	9.2 ± 15.7	0.309	5.9 ± 11.3	6.9 ± 14.3	0.946	10.0 ± 15.7	7.0 ± 14.5	0.230	9.3 ± 15.9	6.9 ± 13.8	0.281
Body image	5.9 ± 14.0	6.9 ± 15.8	0.794	2.0 ± 7.9	3.9 ± 10.8	0.228	3.4 ± 10.2	3.4 ± 10.2	1.000	4.9 ± 11.9	4.9 ± 13.2	0.834	3.4 ± 11.7	2.5 ± 8.8	0.737
Hair loss	5.4 ± 10.9	6.1 ± 12.5	0.738	2.2 ± 8.6	2.9 ± 9.9	0.737	3.7 ± 10.3	2.2 ± 8.6	0.269	0.74 ± 4.5	2.0 ± 6.8	0.15	2.5 ± 9.7	2.0 ± 9.3	0.525

The dysphagia, pain, reflux symptoms and eating restrictions became better from the postoperative 3 months to the postoperative 1 year. However, these trends were not observed in other scales. The multivariable analyses of variance also demonstrated no interaction between the time trend and grouping (Fig. S2, supporting information).

### Endoscopic findings

No significant differences were found in terms of food residual and bile reflux between the two groups. However, the severity of remnant gastritis was milder significantly in the R-Y group, compared with the B-I group. There was only 32.6% of patients in the B-I group suffering from gastritis less than grade 2. The corresponding percentage in the R-Y group was 58.7% (Table 5).

**Table 5:** The results of endoscopic evaluation at postoperative 12 months according to the “residue, gastritis, bile” classification.

	B-I group N = 46 (%)	R-Y group N = 46 (%)	P Value
**Food Residual**			0.749
Grade 0	34 (73.9)	33 (71.7)	
Grade 1	5 (10.9)	4 (8.7)	
Grade 2	2 (4.3)	3 (6.5)	
Grade 3	4 (8.7)	4 (8.7)	
Grade 4	1 (2.2)	2 (4.3)	
**Degree of Gastritis**			0.025
Grade 0	8 (17.4)	19 (41.3)	
Grade 1	7 (15.2)	8 (17.4)	
Grade 2	19 (41.3)	10 (21.7)	
Grade 3	7 (15.2)	4 (8.7)	
Grade 4	5 (10.9)	5 (10.9)	
**Extent of Gastritis**			0.016
Grade 0	8 (17.4)	19 (41.3)	
Grade 1	7 (15.2)	7 (15.2)	
Grade 2	25 (54.3)	16 (34.8)	
Grade 3	6 (13.0)	4 (8.7)	
**Bile reflux**			0.137
Grade 0	37 (80.4)	42 (91.3)	
Grade 1	9 (19.6)	4 (8.7)	

## Discussion

Our results showed that there was no perioperative mortality in either group, and the morbidity and postoperative recovery were comparable between the two groups. However, the estimated blood loss and surgical duration were significantly less in the B-I group, which was accordance with the previous reports. One multicenter RCT in Japan found that there was 34 minutes longer of average operation time in the R-Y group than B-I group, but the overall morbidity were not significantly different (13.6% vs. 8.6%)^13^. Kitagami *et al*. also observed the similar results in the cohorts with laparoscopic distal gastrectomy^16^. The technical-simplicity of B-I anastomosis, which has only one anastomosis and the exemption of duodenal stump handling compared with R-Y group, might attribute to the less blood loss and operation time. However, Ishikawa and Kojima *et al*. demonstrated that there were no significant differences in terms of operation time and blood loss between the two groups, either in open surgery or laparoscopic surgery^11, 15^. These discrepancies could be partly explained by the different operative habits of surgeons, procedures of anastomoses (e.g. hand-sewn or stapling) and surgical instruments (e.g. linear or circular stapler). Some previous researches pointed out that there might be a higher possibility of anastomotic leakage in the B-I anastomosis^13, 15, 25^, which was associated with the impaired blood supply of duodenal stump and excessive tension of anastomotic site^15, 25^. However, there were no anastomosis related complications in the present study. Two cases of intraperitoneal infections were observed in the R-Y group, which might be caused by a longer exposure of digestive tract since longer operation time and more anastomosis were needed.

The R-Y stasis syndrome, which is characterized by the stasis symptoms of upper gastrointestinal tract after R-Y anastomosis, might be induced by an ectopic pace which arises from the proximal part of the Roux limb after the division of small intestine and separates the normal pace from the duodenum^26, 27^. Ishikawa *et al*. found that 20.8% of patients in the R-Y group developed stasis in the early postoperative period^11^. Otsuka *et al*. also demonstrated that 11.6% of patients experienced R-Y stasis syndrome^28^. Therefore, some surgeons advocated to perform the uncut R-Y anastomosis, in order to keep the continuity of digestive tract and decrease the incidence of R-Y stasis syndrome^29, 30^. Nevertheless, the results remained inconsistent even though the uncut R-Y anastomosis was performed. The reason might be not only the continuity of digestive tract, but also the vagus nerve was divided in the operation for gastric cancer. In our study, however, there was only 1 patient suffering from stasis in each group and no significant differences were found in terms of food residual between the two groups, which was consistent with other reports^15^.

Quality of life has been regarded as an important outcome measurement parameter and emphasized in cancer patients. If the same oncological outcomes could be achieved, the postoperative QoL would be an influence factor of the selection of surgical procedures. The clinical practicability and validity of EORTC QLQ-C30 and stomach module QLQ-STO22 questionnaires to assess the QoL of patients with gastric cancer have been proven in many studies^22, 31–33^, including the Chinese version of QLQ-C30 and QLQ-STO22 for Chinese patients^32, 33^.

The present study found that physical functioning, role functioning and global health improved while pain, nausea and vomiting, dysphagia, pain, reflux symptoms and eating restrictions attenuated from the postoperative 3 months to 1 year. Furthermore, these variations accompanied with the time trend did not depend on the grouping. These results suggested that the removal of tumor could improve the QoL and alleviate the digestive symptoms. Moreover, these improvements were independent on the anastomotic methods, which was supported by the findings of Katagami’s study^16^. At the same time, our study showed that the change of QoL could continue to the postoperative 1 year even longer. Nakamura *et al*. also found that symptom scales at 12 months were not significantly different, but were significantly better in the B-I group at 36 months after gastrectomy^34^. These were different from the previous studies indicating that the postoperative 6 months was a cut-point of QoL^35, 36^.

Regarding the reflux symptom, many studies have showed that R-Y anastomosis could reduce the reflux symptom and reflux-related gastritis and esophagitis, and improve the QoL accordingly^8, 11, 12, 15, 37–39^. In this study, we also noticed a decreased incidence of reflux symptom in the R-Y group at postoperative 6 months and 9 months. As time went by, however, the differences of QoL between the two groups diminished. Therefore, there were no statistical differences between the two groups at postoperative 1 year although still better in the R-Y group. In fact, the endoscopic examination found no significant difference in terms of reflux between the two groups at postoperative 1 year. Nevertheless, the degree and extent of remnant gastritis were milder in the R-Y group since more severe reflux has already existed in the B-I group for a long time.

Our results showed that the R-Y group had a marginal better control of dyspnea symptom (P = 0.053). The Japanese RCT also found the superior dyspnea symptom scale in the R-Y group^14^. However, the authors considered that this symptom seemed to be physiologically unrelated to postoperative complications^14^. Insomnia was also considered more as psychological issues than physiological ones^40^. This may be one of the reasons why the scores of insomnia item fluctuated.

Pain is a kind of subjective feeling, which is influenced by many factors, such as postoperative intraperitoneal adhesions^20^, the incidence and severity of reflux symptom and cholelithiasis *etc*. Mathias *et al*. demonstrated that the pain symptoms was the secondary to a defect in motor function of Roux limb and could be observed almost in all the patients undergoing R-Y reconstruction^9^. Takiguchi *et al*. showed that there was no significant difference in terms of pain scale until postoperative 3 years between the two kinds of anastomoses^14^. Nunobe *et al*. also reported that no significant difference on pain was noted between the B-I group and R-Y group at postoperative 5 years^37^. However, the present study found that R-Y anastomosis could result in milder pain significantly at postoperative 1 year, which might benefit from the alleviated reflux-related remnant gastritis. Decreased reflux and pain could contribute to a better global health status at postoperative 1 year in the R-Y group.

There are some limitations of this study. Firstly, the QoL data was obtained by questionnaires, which was a kind of subjective data and easy to be biased by patients’ feeling, cultural background and educational level. Although inevitable in the studies about QoL, using questionnaires to evaluate the QoL is an internationally accepted research approach. Secondly, our interim analysis only reported the 1 year results of this study. We would continue to follow up the patients and evaluate the long-term QoL. Furthermore, more outcome measurements including anemia, stone of gallbladder and long-term survival would be reported in the future. Finally, although being a popular reconstruction method after distal gastrectomy, the Billroth-II anastomosis was considered to be inferior to its counterparts in terms of the postoperative complications, anti-reflux capability and potential risk of remnant gastric cancer^8, 39^. Therefore, the Billroth-II anastomosis was not considered in the design of the trial.

In conclusion, both B-I and R-Y anastomosis are safe and feasible which could be applied in the clinical practice. The stronger anti-reflux capability of R-Y anastomosis contributes to a higher QoL by reducing the reflux related gastritis and pain symptoms, and promoting a better global health.

## Electronic supplementary material

Supplementary Information
