# Author Correction: Comparison of quality of life between Billroth-І and Roux-en-Y anastomosis after distal gastrectomy for gastric cancer: A randomized controlled trial

**DOI:** 10.1038/s41598-018-24612-8

**Published:** 2018-04-25

**Authors:** Kun Yang, Wei-Han Zhang, Kai Liu, Xin-Zu Chen, Zong-Guang Zhou, Jian-Kun Hu

**Affiliations:** 1 Department of Gastrointestinal Surgery, West China Hospital, Sichuan University, Chengdu, China; 2 Laboratory of Gastric Cancer, State Key Laboratory of Biotherapy/Collaborative Innovation Center of Biotherapy and Cancer Center, West China Hospital, Sichuan University, Chengdu, China

Correction to: *Scientific Reports* 10.1038/s41598-017-09676-2, published online 12 September 2017

This Article contains errors in Figure 1.

“Patient demand Roux-en-Y (n = 6)”

should read:

“Patient converted to Roux-en-Y (n = 6)”.

In addition,

“Patient demand Billroth-I (n = 6)”

should read:

“Patient converted to Billroth-I (n = 6)”.

The correct Figure 1 appears below.

**Figure 1:**
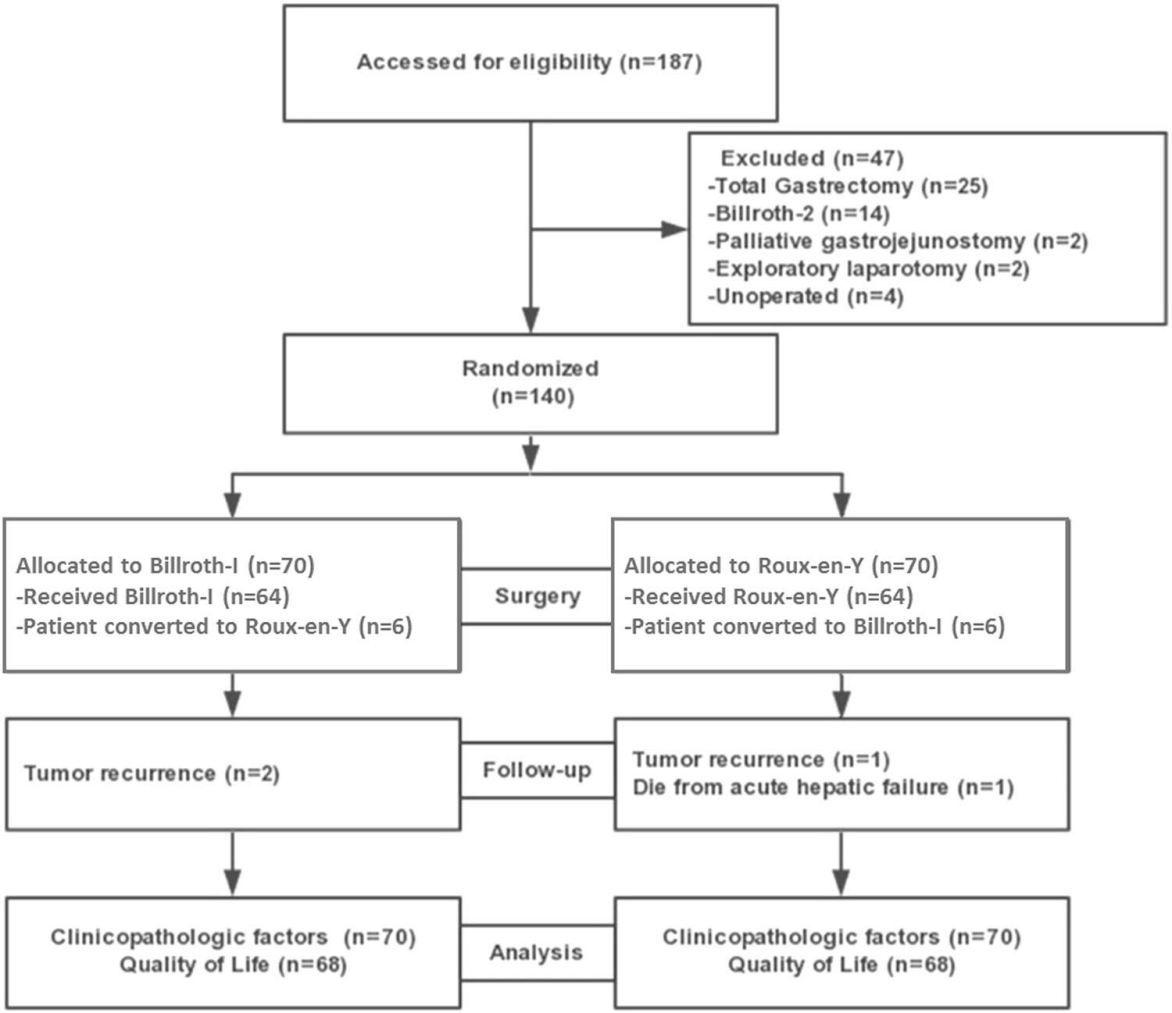
Trial profile.

