# Supplementary material for: Comparison of quality of life between Billroth-І and Roux-en-Y anastomosis after distal gastrectomy for gastric cancer: A randomized controlled trial
Source: Sci Rep. 2017 Sep 12;7:11245. doi: 10.1038/s41598-017-09676-2 (PMC5595810; doi:10.1038/s41598-017-09676-2)
Supplement: Supplementary file 1 — Supplementary Information [file 41598_2017_9676_MOESM1_ESM.pdf]

Comparison of quality of life between Billroth-I and Roux-en-Y anastomosis after distal gastrectomy for gastric cancer: A randomized controlled trial

Kun Yang[1,2#], MD, Wei-Han Zhang[1,2#], MD, Kai Liu[1,2], MD, Xin-Zu Chen[1,2], MD, MD, Zong-Guang Zhou[1], MD, FACS, Jian-Kun Hu[1,2], MD&Ph.D, FRCS

1. Department of Gastrointestinal Surgery, West China Hospital, Sichuan University, China.
2. Institute of Gastric cancer, State Key Laboratory of Biotherapy/Collaborative Innovation Center of Biotherapy and Cancer Center, West China Hospital, Sichuan University, China.

# Co-first authorship: Kun Yang and Wei-Han Zhang contributed equally as the co-first authors.

Correspondence to: Prof. Jian-Kun Hu, MD, Ph.D, FRCS, Department of Gastrointestinal Surgery, and Institute of Gastric cancer, State Key Laboratory of Biotherapy/Collaborative Innovation Center of Biotherapy and Cancer Center, West China Hospital, Sichuan University, No. 37 Guo Xue Xiang Street, Chengdu 610041, Sichuan Province, China. E-mail: hujkwch@126.com; Tel: +86-28-85422878; Fax: +86-28-85164047.

Funding sources: Domestic support from (1) National Natural Science Foundation of China (No. 81301867, 81372344); (2) Sichuan Province Youth Science & Technology Innovative Research Team (No. 2015TD0009); (3) 1. 3. 5 project for disciplines of excellence, West China Hospital, Sichuan University; (4) The Scientific Research Program of Public Health Department of Sichuan Province, China (No. 120196)

Running title: B-I versus R-Y

The manuscript was partly presented at the 11th International Gastric Cancer Congress (IGCC 2015), Sao Paulo, Brazil, June 2015.

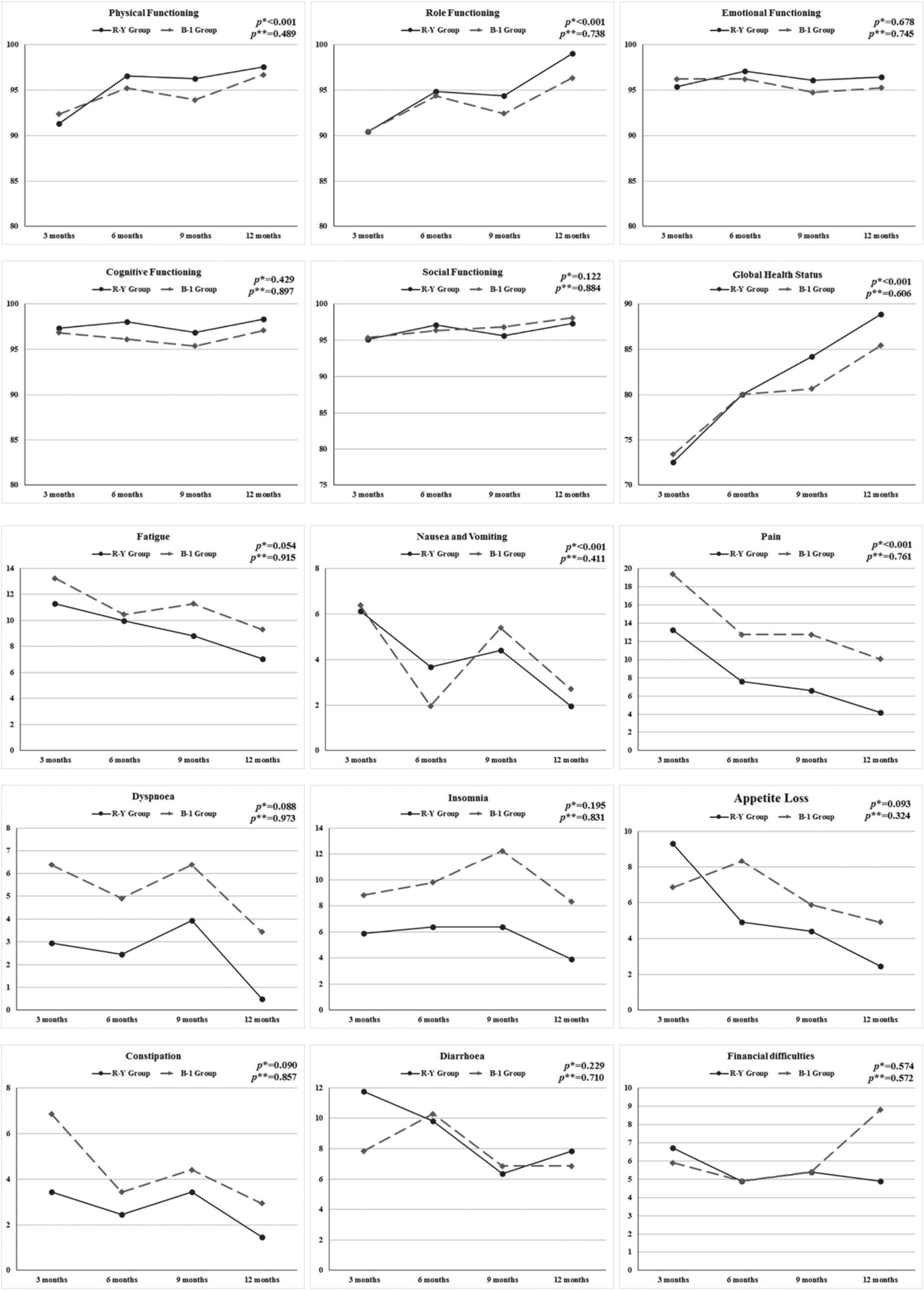

Fig. S1 Change of the postoperative QoL scores according to the QLQ-C30 items. *P**<0.05 means that there were significant variation of each QoL scale accompanied with the time trend. *P***<0.05 means that the variation of each QoL scale accompanied with the time trend depended on the grouping.

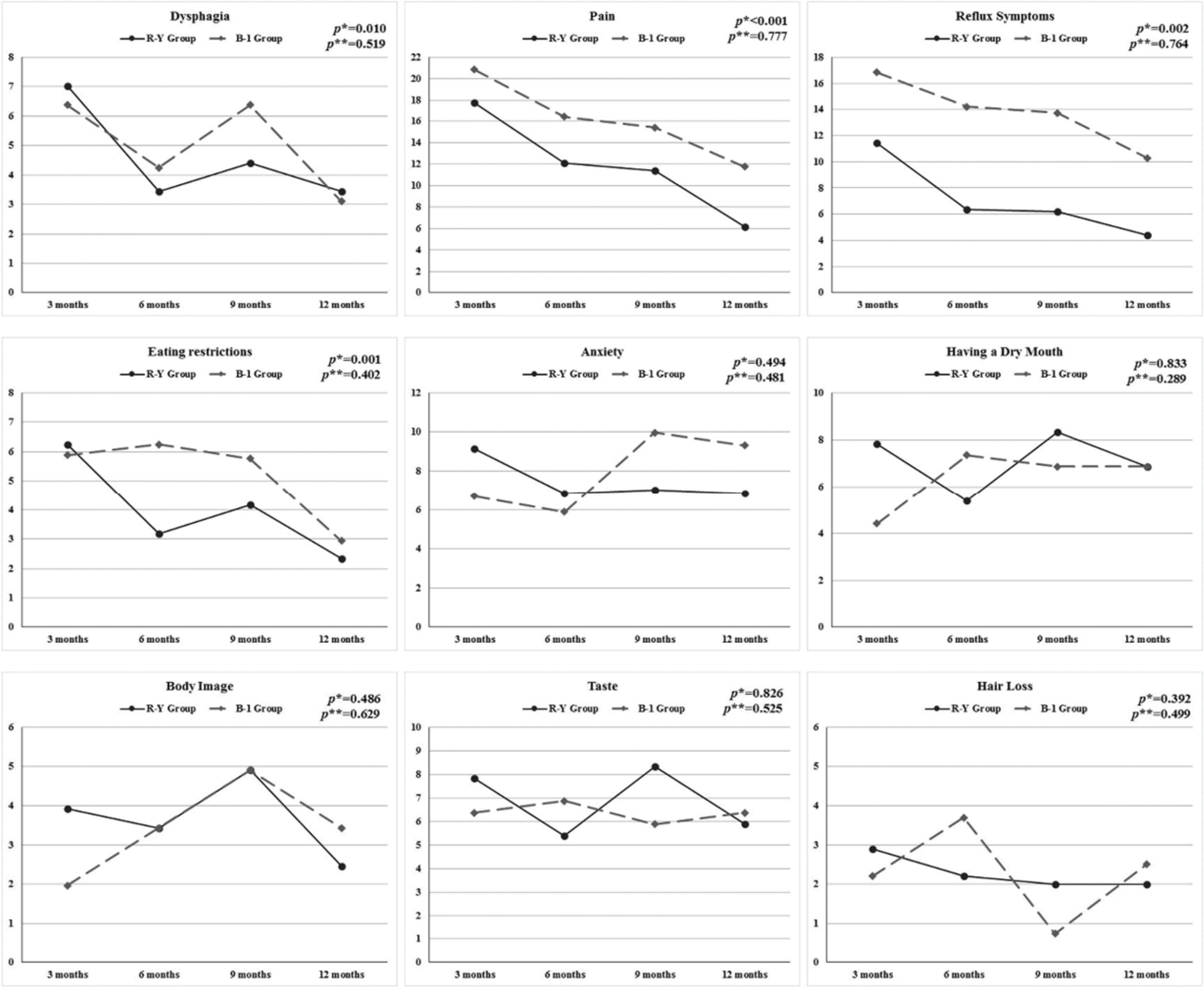

Fig. S2 Change of the postoperative QoL scores according to the QLQ-STO22 items. $P^{*}<0.05$ means that there were significant variation of each QoL scale accompanied with the time trend. $P^{**}<0.05$ means that the variation of each QoL scale accompanied with the time trend depended on the grouping.
